# Early Neurophysiological Abnormalities in Suspected Acute Canine Polyradiculoneuropathy

**DOI:** 10.3390/vetsci11040178

**Published:** 2024-04-16

**Authors:** Laura Porcarelli, Alberto Cauduro, Ezio Bianchi, Claudia Pauciulo, Chiara Maurelli, Daniele Corlazzoli

**Affiliations:** 1 Centro Veterinario Gregorio VII, 00165 Rome, Italy; claudiapauciulo@gregoriovii.com (C.P.); chiaramaurelli@gregoriovii.com (C.M.); danielecorlazzoli@gregoriovii.com (D.C.); 2 Neurovet RSV Legnano, 20025 Legnano, Italy; a.cauduro@neurovet.it; 3 Department of Veterinary Science, University of Parma, 43121 Parma, Italy; ezio.bianchi@unipr.it

**Keywords:** polyradiculoneuritis, ACP, canine, neuromuscular, nerve conduction, electromyography

## Abstract

**Simple Summary:**

Acute polyradiculoneuritis is a common peripheral nerve disorder in dogs, in most cases self-limiting and benign. Electrodiagnostic tests are frequently performed 7 days after the beginning of clinical signs, delaying the correct diagnosis of ACP and other diseases with similar onset. This study aims to retrospectively evaluate 71 dogs with suspected cases of acute polyradiculoneuritis for whom early electrodiagnostic testing (within 6 days) was conducted on some dogs and later testing (7–15 days) on others. Dogs tested later (7–15 days) exhibited more electromyographic abnormalities. Patients tested in the first 6 days exhibited frequent motor nerve conduction and F-wave abnormalities, similar to patients tested between 7 and 15 days. Our results suggest that neurophysiological abnormalities in acute polyradiculoneuritis can be detected within 6 days, enabling an early diagnosis.

**Abstract:**

Acute canine polyradiculoneuritis (ACP) is a common peripheral neuropathy in dogs, and is generally self-limiting and benign. Electrodiagnostic (EDX) tests are typically performed after 7–10 days. Delaying the definitive diagnosis may hamper the treatment of other causes of acute weakness, which may require specific treatments and may carry different prognoses. This retrospective multicenter study aims to assess whether EDX performed within the first 6 days of clinical signs onset can detect alterations indicative of ACP, and aims to characterize the most prevalent alterations. A total of 71 dogs with suspected ACP were retrospectively analyzed and classified into two groups based on EDX timing: early group (EG, 1–6 days after symptom onset) and late group (LG, 7–15 days after symptom onset). In our study, no significant differences were found between the two groups in motor nerve conduction studies (MNCSs) and F-wave analysis, indicating that EDX is able to demonstrate abnormalities even in the first 6 days from onset. Although the LG showed significantly greater degrees of electromyographic (EMG) alterations compared to the EG, frequent muscle alterations were still observed in the EG group. These findings support the use of EDX in patients with suspected ACP within the first 6 days from the clinical onset. Prompt neurophysiological examinations for suspected ACP patients can be performed effectively and can help allow for early diagnosis and facilitate appropriate treatment.

## 1. Introduction

Acute canine polyradiculoneuritis (ACP) is the most common peripheral neuropathy in dogs [1,2,3,4]. Because of its resemblance in clinical presentation and pathological findings to the human autoimmune neuropathy Guillain–Barré syndrome (GBS), it is regarded as the canine counterpart of GBS [1,5,6]. ACP is characterized by progressive weakness, generally affecting the hind limbs first, then progressing to the front limbs, with difficulty maintaining weight and head carriage. Dysphonia is a frequent finding, while megaesophagus is rare. In severe cases, respiratory difficulties may occur [1,4,5,6,7,8,9,10]. Generally, flaccid tetraparesis is observed within one week. Progressive improvement in clinical conditions is observed, often leading to complete recovery over several weeks to months [4,11,12]. The prognosis is guarded in patients developing respiratory difficulties.

ACP etiopathogenesis is not yet fully understood, but it is suspected that an immune-mediated pathology may be triggered by a series of stimuli, including vaccines, infectious agents, and raccoon saliva [13,14]. Histopathological diagnosis is possible through a biopsy of the ventral nerve roots, though this testing is rarely pursued antemortem in dogs. Biopsy results show an inflammatory infiltrate within the ventral nerve roots sampled. Recent studies have demonstrated the presence of serum anti-ganglioside antibodies in patients with ACP, but this test is not currently available in clinical practice [8,15].

ACP diagnosis is currently based on clinical presentation and progression, as well as unremarkable laboratory data, and is supported by electrodiagnostic (EDX) and cerebral spinal fluid (CSF) analysis, following the exclusion of other clinically similar causes of acute weakness, including botulism, acute fulminant myasthenia, tick paralysis, myopathies, and organophosphate toxicity [1,3,9].

Spontaneous muscle activity, secondary to acute axonal damage, is reportedly first observed between 5 and 7 days following injury to the motor axon. Hence, to highlight the maximum number of alterations, the electrodiagnostic (EDX) tests are usually conducted 7–10 days after the onset of symptoms [16,17,18]. This delay in diagnosis could lead to a failure to promptly identify ACP and other acute diseases. Early identification of these conditions is crucial because their treatments and prognoses differ significantly. A previous study [11] reported some electrodiagnostic alterations, including electromyographic (EMG) abnormalities, reduced compound muscle action potential (CMAP) amplitudes, and prolonged F-wave latencies, in ACP patients within the first 4 days following the onset of neurologic signs. One aim of this retrospective study is to assess whether EDX performed early in the disease course (within six days from the onset of symptoms) can detect neurophysiological changes in suspected ACP patients, and a second aim is to describe any EDX alterations detected.

## 2. Materials and Methods

### 2.1. Case Selection

Dogs were recruited from three specialized veterinary centers (Centro Veterinario Gregorio VII—Roma, Neurovet RSV—Legnano, and the Department of Veterinary Science, University of Parma).

Cases were included if they met the following criteria:

- They had a comprehensive neurological examination conducted by an ECVN resident or an ECVN diplomate.
- They had a history of acute onset of neurological signs with progression to tetraparesis.
- Clinical signs were characterized by tetraparesis or tetraplegia with signs of lower motoneuron involvement in all four limbs (hyporeflexia and hypotonia).
- Complete blood count and biochemical tests showed no abnormalities that could explain the symptoms
- Comprehensive EDX testing, including EMG of appendicular muscles, motor nerve conduction studies (MNCSs), F-wave analysis, repetitive nerve stimulation (RNS), sensory nerve conduction studies (SNCs), and cord dorsum potentials (CDPs), were performed between days 1 and 15 days after the onset of symptoms. All repetitive nerve stimulation testing was within normal limits for study inclusion. Patients were also included if they did not undergo repetitive nerve stimulation, provided that EDX abnormalities were so pronounced that RNS was deemed unnecessary, as they were not consistent with a diagnosis of myasthenia and botulism.
- Significant clinical improvement was observed within a six-month period without the use of specific medical interventions such as steroids, NSAIDs, or pyridostigmine. Supportive care, immunoglobulin treatment, and physiotherapy were frequently administered during this time [9].
- Deceased patients or those euthanized due to respiratory difficulties compatible with paralysis of the intercostal and diaphragm muscles were also included, provided they met all the previous criteria (clinical signs, blood tests, and electrodiagnostic test).

Information pertaining to age, breed, weight, gender, ancillary tests, time from symptoms onset and EDX, treatment, physiotherapy, neurological signs, and EDX findings were collected (Table 1).

The information regarding the outcome was obtained from the medical records and through telephone contact with both clients and veterinarians.

Patients were excluded if they had a history of traveling abroad (to avoid the possibility of infestation by non-native ticks), or if they were diagnosed with other neuromuscular diseases (such as myasthenia gravis or polymyositis), diabetes, neoplastic disorders, or recent intoxication. Exclusion criteria also included the presence of dysphagia, inability to wag the tail, megaesophagus, abnormal urinary or fecal control, autonomic signs, regurgitation, and cranial nerve deficits (except palpebral reflex) [9].

Some patients also underwent additional investigations to eliminate other potential causes of muscle weakness, including total body computed tomography (CT) scans, abdominal ultrasounds, MRIs of the spinal cord, thyroid profiles, and serological screening for Toxoplasma, Neospora, Ehrlichia, and Leishmania. Moreover, muscle biopsies were performed in some cases (Table 2).

All clinical data and details about the animal population involved in this study were utilized in compliance with the informed consent statement signed by the owners during the clinical evaluation.

### 2.2. Electrodiagnostic Study (EDX)

Electrodiagnostic studies were performed under general inhalation anesthetic protocol. The neurophysiological tests were performed using standard procedures [16] by an experienced ECVN resident (LP) supervised by an ECVN diplomate (DC), and by 2 ECVN diplomates (EB and AC).

The neurophysiological tests were carried out with standard EMG instruments: an UltraPro S100 (NATUS Medical Incorporated, Pleasanton, CA, USA), Myoquick (Micromed SpA, Treviso, Italy), Nemus2 (EB Neuro SpA., Florence, Italy), and Easyneuro (Omicron T srl, Naples, Italy).

The patients were divided into two groups based on whether they underwent the EDX between 1 and 6 days from the onset of symptoms (early group, EG) or between 7 and 15 days (late group, LG).

In veterinary medicine, the most reliable electrophysiological indicators of ACP are the presence of spontaneous pathological activity at EMG, decreased compound muscle action potential (CMAP) amplitudes at motor nerve conduction studies (MNCSs), increased minimum F-wave latencies, increased F Ratios, and decreased or absent F-wave amplitudes [1]. Based on these diagnostic criteria, we compared the results of the electrophysiological study between the early group and the late group to assess the possibility of making a diagnosis of ACP in the early stages of the disease.

The following parameters were recorded:

#### 2.2.1. Electromyography (EMG)

A disposable bipolar concentric needle electrode and a subcutaneous ground electrode were used for EMG analysis.

Electromyography was performed, recording spontaneous activity from the supraspinatus, infraspinatus, triceps brachii, biceps brachii, extensor carpi radialis, thoracic limbs flexors, palmar interosseus, gluteal, vastus lateralis, semitendinosus, tibialis cranialis, gastrocnemius, and plantar interosseus muscles.

**Figure 1:**
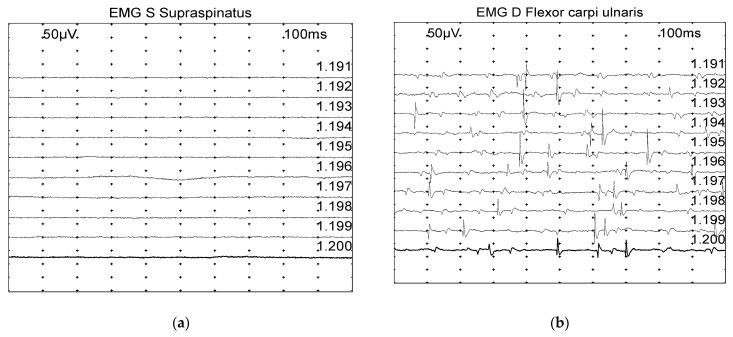

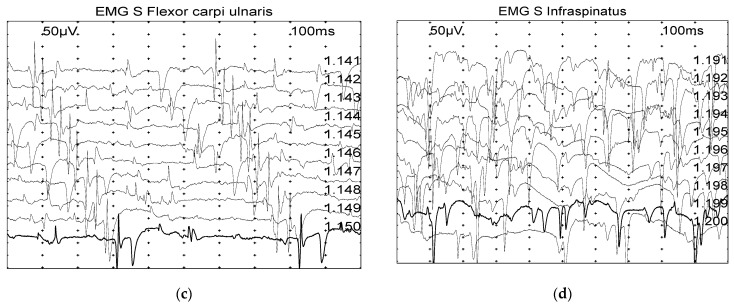
Examples of spontaneous muscle activity graded on a scale from 0 to 4, with the grade also depending on the number of involved areas: (**a**) Grade 0: normal. (**b**) Grade 2: moderate number of fibrillation potentials/positive sharp wave. (**c**) Grade 3: several fibrillation potentials and positive sharp wave. (**d**) Grade 4: full interference pattern of potentials.

Spontaneous activity (positive sharp waves and fibrillation potentials) was graded based on a scale from 0 to 4 (Figure 1) [17]:

- 0: none found
- 1+: Persistent single trains of potential (>2–3 s) in at least two areas
- 2+: Moderate number of potentials at least in 3 areas
- 3+: Several potentials in all areas
- 4+: Full interference pattern of potentials.

#### 2.2.2. Motor Nerve Conduction Studies (MNCSs)

The motor nerve conduction studies (MNCSs) involved orthodromic stimulation of the ulnar and sciatic nerves, followed by recording the resulting compound muscle action potentials (CMAPs).

For stimulation, stainless-steel monopolar electrodes coated with polytetrafluoroethylene of various lengths featuring 2–3 mm uncovered tips were employed. Stainless-steel monopolar electrodes were used for recording, and a ground electrode was placed subcutaneously between the stimulation and recording electrodes.

The CMAP was elicited with supramaximal stimuli lasting 0.2 ms. CMAP amplitude was measured from the largest negative peak to the largest positive peak. Recording of CMAP occurred in the palmar interosseous muscles following stimulation of the ulnar nerve at the level of the elbow and carpus and in the plantar interosseous muscles after stimulating the sciatic–tibial nerve at the level of the sciatic notch and hock, as previously described [16,18].

The values of CMAP amplitude, Motor nerve conduction velocity (MNCV), conduction block (CB), and temporal dispersion (TD) were assessed. Conduction block was defined by >50% reduction in CMAP amplitude when stimulated proximally as compared to distally [16].

#### 2.2.3. F-Wave

The F-wave was obtained after stimulation at the level of carpus for the ulnar nerve and at the level of the hock for the sciatic–tibial nerve, using supramaximal stimuli of 0.2 ms duration. The parameters evaluated included F-wave detectability, F-wave minimum latency, and F Ratio.

The expected F latency was calculated using the equations:

- Ulnar nerve expected minimum F-wave latency (ms) [16,19]:
  6.03 + 0.22 × limb length (cm).(1)
- Sciatic–tibial nerve expected minimum F-wave latency (ms) [16,19]:
  3.45 + 0.33 × limb length (cm).(2)

The F Ratio was calculated using the equation [20]:

(F latency − M latency − 1)/(2 × M latency).(3)

#### 2.2.4. Sensory Nerve Conduction Studies (SNCs) and Cord Dorsum Potentials (CDPs)

For sensory nerve conduction (SNC) recording, electrical stimulation was applied as a rectangular wave of 0.2 ms duration with the maximal possible intensity without motor interference. The sensory nerve action potential (SNAP) amplitude was recorded from the largest negative peak to the largest positive peak. The values of sensory nerve conduction velocity (SNCV) and cord dorsum Potentials were assessed. The nerves tested were radial, ulnar, or tibial nerves as previously described [16,21,22].

#### 2.2.5. Repetitive Nerve Stimulation

Repeated supramaximal stimulation was conducted on the sciatic–tibial and ulnar nerves using sets of 10 supramaximal stimuli, each lasting 0.2 ms for every stimulation rate. These stimuli were administered at frequencies of 2 Hz and 3 Hz. The amplitude of CMAP and the area under the curve were then compared across the initial, fifth, and tenth potentials to evaluate decremental responses [16,23].

### 2.3. Statistical Analysis

The distribution of parameters was presented in numbers and percentages. The normality of continuous variables was measured using the Shapiro-Wilk test, and depending on the data normality, a two-sided *t*-test or Wilcoxon rank-sum (Mann–Whitney U) test was applied. Nominal and ordinal outcomes were compared using the chi-square and Wilcoxon rank-sum (Mann–Whitney U) tests, respectively. The significance was set to a *p*-value of 0.05.

All statistical analysis was performed with STATA 18.0 statistical software (StataCorp 2023, College Station, TX, USA).

## 3. Results

A total of 71 cases diagnosed with suspected ACP were analyzed, with 33 patients from the University of Parma, 23 patients from Centro Veterinario Gregorio VII, and 15 patients from Neurovet RSV. No statistically significant differences between the two groups were noted regarding the patients’ origin. Dogs included in this study comprised 28 breeds, mongrel being the most common breed in both groups (*n* = 24, 33.8%). The average age of patients was 95.0 ± 41.2 months. Female dogs (*n* = 37, 52.1%) were slightly more represented than the males (*n* = 34, 47.9%). Groups did not differ in breed, average age, median weight, or gender of patients (Table 1).

Two-thirds of patients (*n* = 47, 66.2%) underwent EDX within 7 to 15 days after the symptom onset with an average of 9.9 ± 2.7 days (late group, LG), while EDX tests for the remaining patients (*n* = 24, 33.8%) were performed during the first week (1 to 6 days, on average 4.0 ± 1.2 days, early group, EG).

Patients’ signs severity was similar in both groups, with non-ambulatory tetraparesis with four limbs hyporeflexia being the most common form (*n* = 45, 63.4%) (Table 2).

For diagnostic purposes, every patient was tested for complete blood count and blood chemistry. Additionally, abdominal ultrasound, thorax X-ray, MRI imaging, total body CT, serological analysis, thyroid panels, botulin test, CSF analysis, echocardiography, electrophoresis, and muscle biopsy were tested in some cases (Table 2). There were no significant differences in the distribution of diagnostic methods used between the groups for all tests, except for serology, which was only applied in the late diagnosis group (*p* = 0.046).

The majority of patients in both groups were treated with physiotherapy (*n* = 40, 56.3%) or a combination of physiotherapy and vitamin B and L-carnitine (*n* = 22, 31.0%). Overall, there was no statistical difference between the groups’ treatment allocation (Table 2).

During the follow-up period, the great majority of patients showed improvement in up to 9 weeks and returned to normal (*n* = 65, 91.5%). Six patients underwent euthanasia due to respiratory difficulties (8.5%). Overall, no statistical difference was observed among the groups regarding the follow-up outcome (Table 2).

### 3.1. Electrodiagnostic Results

Every patient in both the early and late groups exhibited at least one abnormality in the electrodiagnostic study (EMG, MNCS, F-wave studies, SNCs, and CDP).

#### 3.1.1. Electromyography (EMG)—Late Diagnostic Group Is Associated with Greater EMG Alterations

The electromyographic study did not show alterations in the muscles of the upper limbs in 1 out of 24 patients (4.2%) and in the muscles of the lower limbs in another patient (1 out of 24, 4.2%) in the early group. In comparison, all patients in the late group exhibited at least one electromyographic abnormality in both the upper and lower limbs.

The results from the EMG analysis measured with a scale ranging from zero to 4, where zero represents normality and 4 represents greater alteration, found that patients in the late group show significantly higher rates of pathologic spontaneous muscle activity (positive sharp waves and fibrillation potentials) (Table 3). This significance was observed in all muscles except for the infraspinatus. The high number of missing data on this muscle may explain the finding.

#### 3.1.2. Motor Nerve Conduction Studies (MNCSs)

A motor nerve conduction study was performed on two nerves: the ulnar and sciatic–tibial nerves.

Most patients showed at least one alteration in the motor nerve conduction studies (Table 4). In the early group, 75% of patients had alterations in the ulnar nerve and 95.8% in the sciatic–tibial nerve, while in the late group, 87.7% of patients had alterations in the ulnar and 87.2% in the sciatic–tibial nerve. No significant difference between the groups was noted. The most frequent alteration was a decrease in CMAP amplitude by 58% for the ulnar nerve and 83% for the sciatic–tibial nerve for the EG and 53% for the ulnar nerve and 63% for the sciatic–tibial nerve for the LG.

The only statistical significance was observed in the decrease in MNCV (*p* = 0.009) of the ulnar nerve between the early and late groups.

#### 3.1.3. F-Wave

The F-wave was not evocable for the ulnar nerve in 6 (25%) and 10 (21.3%) cases in the EG and the LG, respectively. Similarly, the F-wave was not recorded in the sciatic–tibial nerves of 9 (37.5%) and 12 (25.5%) patients in the EG and the LG, respectively. In patients in whom the F-wave could be recorded, the most commonly observed alteration in both groups was an increase in the latency of the F-wave. No significant difference in F-wave studies was observed between the two groups (Table 5).

#### 3.1.4. Sensory Nerve Conduction Studies (SNCs) and Cord Dorsum (CDP)

A total of 16.7% of the cases in the EG and 23.4% of the patients in the LG exhibited at least one abnormality in either sensory nerve conduction studies (SNCs) or cord dorsum potentials. Reduced SNAP amplitude was present in 8.3% in EG and 10.6% in LG, while reduced SNCV was present in 8.3% in EG and 12.8% in LG. These parameters were not significantly different between the study groups. Cord dorsum potentials showed abnormalities in amplitude or latency in 19.2% of patients only in the LG. All patients who showed abnormalities at CDP had abnormalities in the SNCs.

#### 3.1.5. Repetitive Nerve Stimulation

The repetitive nerve stimulation was not performed in 8/71 (11.3%) of patients. In the remaining 63/71 (88.7%), the test was within the normal limits.

## 4. Discussion

The purpose of our study was to evaluate whether EDX performed within the first six days allows diagnosis of suspected ACP cases in dogs.

In our study, it was possible to identify at least one EDX alteration in all patients, including EG patients. Seventy out of seventy-one patients (98.6%) exhibited abnormalities in both EMG and MNCS. Only one patient in the LG showed EMG abnormalities and normal MNCS; this patient showed rapid and spontaneous improvement with physiotherapy alone. Although the alterations were more severe in the late group, we were able to diagnose ACP in all patients and rule out other differential diagnoses, such as acute fulminant myasthenia [16,24].

LG patients showed a statistically significant difference in the degree of EMG alterations compared to early group patients, according to the previously published literature [11]. Despite this, the early group patients exhibited frequent alterations in most of the muscles examined, both in the anterior and posterior limbs. This finding is in accordance with a previous study [11].

Changes in MNCS were found in a high percentage of EG cases, with the most common alteration being the reduction in CMAP amplitudes in the ulnar and sciatic–tibial nerves. This finding is consistent with previous studies and reflects a greater prevalence of axonal alterations in dogs affected by ACP compared to human patients with Guillain–Barre syndrome [25]. Some alterations consistent with demyelinating pathology (reduction in MNCV and temporal dispersion) are, however, frequent in both groups.

Interestingly, our data did not show a significant difference between the early and late groups in the analysis of motor nerve conduction studies. The only significant difference between the EG and LG in the analysis of MNCS was a decreased MNCV in the ulnar nerve in the LG. The reduction in MNCV is consistent with demyelinating processes. This difference between the two groups could be attributed to the natural progression of ACP, where demyelination represents a later event compared to axonopathy [24,25]. Since in ACP, the ulnar nerve is involved later than the tibial sciatic nerve, we could assume that in the EG the ulnar nerve is exhibiting electrophysiological features of axonopathy (reduction in CMAP amplitude) but not yet signs of demyelination (reduction in MNCV), which are instead evident in the LG. The evolution of the disease also justifies the lack of a significant difference between the EG and the LG in the analysis of the MNCV of the sciatic–tibial nerve. The sciatic–tibial nerve is involved early in the disease process and alterations related to both axonopathy and demyelination are evident in the EG, so the reduction in MNCV was observed in both groups. Nevertheless, it is important to interpret this result cautiously due to the limited number of patients.

The F-wave abnormalities in ACP are very common, especially on the sciatic–tibial nerve [11]. Our findings also showed more alterations in the sciatic nerve in a great majority of participants, although the timing of diagnosis did not affect the distribution of F-wave abnormalities in the study groups.

F-wave abnormalities in our study are consistent with those reported in the literature. Increased latency, absence of the F-wave, and an elevated F Ratio are commonly observed [9,11]. These alterations indicate a more significant demyelinating component in the nerve root compared to more distal portions of motor nerves, where axonal alterations are more prominent [11].

In our study, the motor nerves were more affected than the sensory nerves both in the early and late stages. These data are in accordance with the literature, which reports minimal involvement of the sensory component compared to the motor component [11]. The abnormalities of CDP were present only in the LG, but this finding should be taken with caution due to the small number of patients. The abnormalities detected in the CDP were present only in patients with abnormalities in the SNCs. This finding is attributable to the fact that lesions of sensory nerves distal to the root ganglia result in CDP alterations dependent on the severity of the lesions [26].

In human medicine, electrodiagnostic studies conducted within the first week of symptom onset show a lower percentage of abnormalities compared to our study [27]. A possible explanation for this discrepancy is that in humans, the predominant form of GBS is demyelinating. Neurophysiological and pathological findings indicate that demyelination of motor nerves is focal and patchy and the probability of finding abnormal results increases with the number of nerves studied [28]. In dogs, the predominant histopathological pattern is mixed or axonal, hence EMG abnormalities (i.e., fibrillation potentials and positive sharp waves) and decreased CMAP amplitude are more likely detected compared to humans [11,25,29]. Another possible explanation for less evident neurophysiological alterations in the early stages in humans is that the progression of the pathology is slower, with the progressive phase lasting four weeks [8,30]. As a consequence, in humans, the greatest EDX alterations are expected after 15 days, while EDX abnormalities can be expected earlier in dogs, probably because of a more rapid progression (5–10 days) [1,11].

Neurophysiological studies are essential for diagnosing and differentiating neuromuscular diseases, but the timing of their execution in cases of suspected ACP is controversial. Generally, these tests are not recommended before 7–10 days due to the scarcity of alterations found [31,32,33]. Such delay in diagnosis could lead to the failure to recognize other diseases with similar clinical signs, which may require specific treatment [24]. Moreover, correct and prompt diagnosis of ACP could allow for targeted treatment such as plasma exchange or immunoglobulins and could improve the outcome of the most severe patients or those with respiratory difficulties. In humans, early treatment with plasma exchange or immunoglobulins has been shown to reduce the need for mechanical ventilation and promote faster recovery [34]. Plasma exchange yields benefits if performed within four weeks, although the greatest benefits are evident in patients who are treated early [34]. In veterinary medicine, treatment with immunoglobulins and plasma exchange is seldom utilized. To our knowledge, there is a single study on the use of immunoglobulins in dogs with ACP and a single case report on the use of plasma exchange in a patient with ACP and respiratory difficulties [4,30]. Although their benefit still needs to be demonstrated, early diagnosis might increase the number of patients who will have access to such treatments in more severe cases in the future.

Our study had certain limitations due to its retrospective nature. Clinical data reported by various clinicians were not standardized, potentially resulting in the omission of certain clinical signs during the initial examination, as well as imprecise recording of episode durations. Additionally, some follow-ups were obtained via telephone calls, and this does not permit direct neurological assessments. Moreover, owners may not promptly notice early symptoms of weakness, especially if it is mild. Therefore, accurately indicating the exact onset date of symptoms may be difficult.

Finally, another limitation related to the retrospective nature of the study is the variability of electrodiagnostic data. Not all patients underwent the same testing protocol. The examinations were conducted in three different centers using four different machines that may have different settings and sensitivities. Although the studies were performed using the same described technique and there are no statistically significant differences between the two groups regarding the patients’ origin, it is not possible to exclude variability in the execution of the examinations. Another limitation of the study is the lack of a definitive antemortem test for ACP, and although inclusion and exclusion criteria are designated to limit diagnostic errors, the possibility of misdiagnosis and selection bias remains. The limited number of patients and the retrospective nature of the study could affect statistical power; therefore, prospective studies with a larger sample size and well-standardized inclusion criteria would be necessary to confirm our results.

## 5. Conclusions

In conclusion, our findings suggest that neurophysiological abnormalities can be detected in ACP even in its early stages. Early detection may aid in the timely and appropriate treatment of patients by ruling out more severe neuro-muscular conditions. These findings offer valuable supplementary information for veterinarians and owners alike. Consequently, we strongly recommend prompt neurophysiological examinations for suspected ACP patients.

## Figures and Tables

**Table 1:** Demographic parameters of study participants.

	Early Group (*n* = 24)	Late Group (*n* = 47)	*p*-Value *
Breed (*n*, %)			0.121 ^a^
Mongrel	5 (20.8%)	19 (40.4%)	
German Shepherd	2 (8.3%)	2 (4.3%)	
Labrador Retriever	1 (4.2%)	4 (8.5%)	
Jack Russel Terrier	1 (4.2%)	3 (6.4%)	
Poodle	1 (4.2%)	2 (4.3%)	
Epagneul Breton	1 (4.2%)	1 (2.1%)	
Spitz	1 (4.2%)	1 (2.1%)	
West Highland White Terrier	1 (4.2%)	1 (2.1%)	
Bolognese	2 (8.3%)	-	
Border Collie	2 (8.3%)	-	
Rough Collie	2 (8.3%)	-	
Beagle	1 (4.2%)	-	
Cavalier King Charles Spaniel	1 (4.2%)	-	
Italian Spinone	1 (4.2%)	-	
Berger de Beauce	1 (4.2%)	-	
Springer Spaniel	1 (4.2%)	-	
Cocker Spaniel	-	4 (8.5%)	
Fox Terrier	-	2 (4.3%)	
Golden Retriever	-	2 (4.3%)	
Australian Shepherd	-	1 (2.1%)	
Belgian Shepherd	-	1 (2.1%)	
Chihuahua	-	1 (2.1%)	
Dachshund	-	1 (2.1%)	
Shi-Tzu	-	1 (2.1%)	
Siberian Husky	-	1 (2.1%)	
Age (months) (Mean ± SD)	91.3 ± 36.3	96.9 ± 43.9	0.591 ^b^
Weight (kg) (Median (IQR))	13.25 (7–21.2)	12 (7.3–23.2)	0.971 ^c^
Gender, *n* (%)			0.588 ^a^
Female	10 (41.7%)	16 (34.0%)	
Male	10 (41.7%)	20 (42.6%)	
Spayed Female	2 (8.3%)	9 (19.2%)	
Neutered Male	2 (8.3%)	2 (4.3%)	

* Groups were compared using the following tests: (^a^) chi-square; (^b^) two sample *t*-test; (^c^) Mann–Whitney U.

**Table 2:** Clinical and diagnostic parameters of study participants in the early and late diagnostic groups.

	Early Group *n* = 24, (%)	Late Group *n* = 47, (%)	*p*-Value *
Severity of Symptoms			0.094
Amb. Tetraparesis, hyporeflexia	3 (12.5%)	15 (31.9%)	
Amb. Tetraparesis, hyporeflexia with palpebral deficit	0 (0%)	1 (2.1%)	
Non-amb. Tetraparesis, hyporeflexia	20 (83.3%)	25 (53.2%)	
Non-amb. Tetraparesis, hyporeflexia with palpebral deficit	1 (4.2%)	1 (2.1%)	
Tetraplegia, hyporeflexia	0 (0%)	5 (10.6%)	
Ancillary tests			
Ultrasound	12 (50.0)	17 (36.2)	0.262
Thorax X-ray	14 (58.3)	17 (36.2)	0.075
MRI	2 (8.3)	5 (10.6)	0.758
Total Body CT	0 (0)	2 (4.3)	0.305
Serology	0 (0)	7 (14.9)	0.046
Thyroid panel	1 (4.2)	4 (8.5)	0.499
Botulin test	1 (4.2)	1 (2.1)	0.623
CSF analysis	0 (0)	1 (2.1)	0.472
Echocardiogram	1 (4.2)	0 (0)	0.159
Electrophoresis	0 (0)	1 (2.1)	0.472
Muscle biopsy	0 (0)	1 (2.1)	0.472
Treatment			0.413
Physiotherapy	11 (45.8%)	29 (61.7%)	
Physiotherapy + Vit B + L-carnitine	10 (41.7%)	12 (25.5%)	
Physiotherapy + Immunoglobulins	0 (0%)	1 (2.1%)	
Immunoglobulins	1 (4.2%)	0 (0%)	
Intensive Care	2 (8.3%)	4 (8.5%)	
None	0 (0%)	1 (2.2%)	
Follow-up status			0.980
Return to Normal	22 (91.7%)	43 (91.50%)	
Euthanasia	2 (8.3%)	4 (8.5%)	

* Groups were compared using a Chi-Square test.

**Table 3:** Distribution of scores from the electromyographic studies in the early and late diagnostic group patients.

Muscle	Early Group *n* (%)						Late Group *n* (%)						*p*-Value *
	0	1	2	3	4	N/A	0	1	2	3	4	N/A	
Supraspinatus	16 (66.7)	1 (4.2)	0 (0)	3 (12.5)	2 (8.3)	2 (8.3)	8 (17.0)	8 (17.0)	9 (19.2)	11 (23.4)	3 (6.4)	8 (17.0)	0.004
Infraspinatus	5 (20.8)	3 (12.5)	1 (4.2)	1 (4.2)	2 (8.33)	12 (50.0)	5 (10.6)	2 (4.3)	4 (8.5)	3 (6.4)	6 (12.8)	27 (57.5)	0.173
Triceps Brachii	13 (54.2)	3 (12.5)	1 (4.2)	6 (25.0)	1 (4.2)	0 (0)	5 (10.6)	8 (17.0)	12 (25.5)	11 (23.4)	9 (19.2)	2 (4.3)	0.002
Biceps Brachii	12 (50.0)	4 (16.7)	2 (8.3)	3 (12.5)	3 (12.5)	0 (0)	4 (8.5)	3 (6.4)	13 (27.7)	12 (25.5)	13 (27.7)	2 (4.3)	<0.001
Extensor Carpi Radialis	11 (45.8)	5 (20.8)	3 (12.5)	2 (8.3)	3 (12.5)	0 (0)	5 (10.6)	1 (2.1)	6 (12.8)	16 (34.0)	18 (38.3)	1 (2.1)	<0.001
Thoracic Limb Flexors	7 (29.2)	2 (8.3)	2 (8.3)	2 (8.3)	4 (16.7)	7 (29.2)	4 (8.5)	3 (6.4)	5 (10.6)	9 (19.2)	12 (25.5)	14 (29.8)	0.044
Interosseous	2 (8.3)	6 (25.0)	8 (33.3)	4 (16.7)	4 (16.7)	0 (0)	2 (4.26)	3 (6.4)	4 (8.5)	15 (31.9)	22 (46.8)	1 (2.1)	0.001
Gluteal	17 (70.8)	1 (4.2)	3 (12.5)	0 (0)	3 (12.5)	0 (0)	14 (29.8)	9 (19.2)	6 (12.8)	7 (14.9)	6 (12.8)	5 (10.6)	0.015
Vastus Lateralis	14 (58.3)	1 (4.2)	4 (16.7)	1 (4.2)	2 (8.3)	2 (8.3)	5 (10.6)	7 (14.9)	8 (17.0)	13 (27.6)	6 (12.8)	8 (17.0)	0.001
Semitendinosus	16 (66.7)	1 (4.2)	2 (8.3)	3 (12.5)	0 (0)	2 (8.3)	7 (14.9)	7 (14.9)	11 (23.4)	6 (12.8)	10 (21.3)	6 (12.8)	<0.001
Gastrocnemius	6 (25.0)	6 (25.0)	2 (8.3)	6 (25.0)	4 (16.7)	0 (0)	1 (2.1)	1 (2.1)	12 (25.5)	11 (23.4)	21 (44.7)	1 (2.1)	0.001
Cranial Tibial	8 (33.3)	8 (33.3)	1 (4.2)	6 (25.0)	1 (4.2)	0 (0)	2 (4.3)	2 (4.3)	11 (23.4)	15 (31.9)	16 (34.0)	1 (2.1)	<0.001
Plantaris interosseous	1 (4.2)	7 (29.2)	7 (29.2)	7 (29.2)	2 (8.3)	0 (0)	1 (2.1)	4 (8.5)	5 (10.6)	16 (34.0)	20 (42.6)	1 (2.1)	<0.001

* Groups were compared using the Wilcoxon rank sum test; N/A: Not Applicable.

**Table 4:** Motor Nerve Conduction Study Results.

	Ulnar Nerve			Sciatic–Tibial Nerve		
	Early Group *n* (%)	Late Group *n* (%)	*p*-Value	Early Group *n* (%)	Late Group *n* (%)	*p*-Value
CMAP amplitude abnormalities			0.316			0.089
Yes	14 (58.3)	25 (53.2)		20 (83.3)	30 (63.8)	
No	6 (25.0)	19 (40.4)		4 (16.7)	17 (36.2)	
N/A	4 (16.7)	3 (6.4)		-		
Decrease in MNCV			0.009			0.453
Yes	7 (29.2)	30 (63.8)		10 (41.7)	24 (51.1)	
No	13 (54.2)	13 (27.7)		14 (58.3)	23 (48.9)	
N/A	4 (16.7)	4 (8.5)		-	-	
Conduction Block			0.339			0.085
Yes	5 (20.8)	16 (34.0)		2 (8.3)	12 (25.5)	
No	15 (62.5)	27 (57.5)		22 (91.7)	35 (74.5)	
N/A	4 (16.7)	4 (8.5)		-	-	
Temporal Dispersion			0.39			0.055
Yes	7 (29.2)	20 (42.6)		16 (66.7)	20 (42.6)	
No	13 (54.2)	23 (48.9)		8 (33.3)	27 (57.5)	
N/A	4 (16.7)	4 (8.5)		-	-	

CMAP: Compound Muscle Action Potentials, MNCV: Motor Nerve Conduction Velocity; N/A: Not Applicable. Groups were compared using a Chi-Square test.

**Table 5:** F-wave studies results.

	Ulnar Nerve			Tibial Nerve		
	Early Group *n* (%)	Late Group *n* (%)	*p*-Value	Early Group *n* (%)	Late Group *n* (%)	*p*-Value
F-wave (at least one abnormality)			0.945			0.275
Yes	12 (50.0)	26 (55.3)		21 (87.5)	36 (76.6)	
No	8 (33.3)	18 (38.3)		3 (12.5)	11 (23.4)	
Not performed	4 (16.7)	3 (6.4)		-	-	
F-wave latency abnormalities			0.697			0.603
Yes	6 (25.0)	16 (34.0)		9 (37.5)	17 (36.2)	
No	7 (29.2)	11 (23.4)		5 (20.8)	13 (27.7)	
No F-wave detected	6 (25.0)	10 (21.3)		9 (37.5)	12 (25.5)	
Not performed	5 (20.8)	10 (21.3)		1 (4.2)	5 (10.6)	
Increased F Ratio						0.490
Yes				8 (33.3)	16 (34.0)	
No				7 (29.2)	16 (34.0)	
No F-wave detected				9 (37.5)	12 (25.5)	
Not performed				-	3 (6.4)	

Groups were compared using a Chi-Square test.

## Data Availability

The data presented in this study are available on request from the corresponding author.
